# Priming of *Salmonella enterica* Serovar Typhi-Specific CD8^+^ T Cells by Suicide Dendritic Cell Cross-Presentation in Humans

**DOI:** 10.1371/journal.pone.0005879

**Published:** 2009-06-11

**Authors:** Rosângela Salerno-Goncalves, Marcelo B. Sztein

**Affiliations:** Center for Vaccine Development, University of Maryland School of Medicine, Baltimore, Maryland, United States of America; New York University School of Medicine, United States of America

## Abstract

**Background:**

The emergence of antibiotic-resistant strains of *Salmonella enterica* serovar Typhi (*S*. Typhi), the etiologic agent of typhoid fever, has aggravated an already important public health problem and added new urgency to the development of more effective typhoid vaccines. To this end it is critical to better understand the induction of immunity to *S.* Typhi. CD8^+^ T cells are likely to play an important role in host defense against *S.* Typhi by several effector mechanisms, including killing of infected cells and IFN-γ secretion. However, how *S.* Typhi regulates the development of specific CD8^+^ responses in humans remains unclear. Recent studies in mice have shown that dendritic cells (DC) can either directly (upon uptake and processing of *Salmonella*) or indirectly (by bystander mechanisms) elicit *Salmonella*-specific CD8^+^ T cells.

**Methodology/Principal Findings:**

We report here that upon infection with live *S.* Typhi, human DC produced high levels of pro-inflammatory cytokines IL-6, IL-8 and TNF-α, but low levels of IL-12 p70 and IFN-γ. In contrast, DC co-cultured with *S.* Typhi-infected cells, through suicide cross-presentation, uptake *S.* Typhi-infected human cells and release high levels of IFN-γ and IL-12p70, leading to the subsequent presentation of bacterial antigens and triggering the induction of memory T cells, mostly CD3^+^CD8^+^CD45RA^−^CD62L^−^ effector/memory T cells.

**Conclusions/Significance:**

This study is the first to demonstrate the effect of *S.* Typhi on human DC maturation and on their ability to prime CD8^+^ cells and highlights the significance of these phenomena in eliciting adaptive immunity to *S.* Typhi.

## Introduction

Typhoid fever remains an important public health priority, particularly in developing countries, with an estimated 16 million new cases annually and 600,000 deaths [1]. The emergence of antibiotic-resistant *Salmonella enterica* serovar Typhi (*S.* Typhi), the etiologic agent of typhoid fever, has aggravated an already important public-health problem [2], [3]. *S.* Typhi, is a facultative intracellular bacterial pathogen with the capacity to survive and replicate in phagocitic and non-phagocytic cells [4], [5], [6], [7]. T cells might play an important role in immunity to *S.* Typhi. We have shown in volunteers immunized orally with attenuated strains of *S.* Typhi, including Ty21a, as well as with the novel attenuated typhoid vaccine candidate strains CVD 908, CVD 908-htrA and CVD 909, the induction of CD8^+^ cell-mediated immunity (CMI) mechanisms that involve the secretion of IFN-γ and the killing of *S.* Typhi-infected cells by cytotoxic T lymphocytes [5], [6], [7], [8], [9], [10], [11], [12], [13], [14]. Our group also demonstrated the ability of classical HLA class Ia, as well as the non-classical HLA class Ib molecule HLA-E, to function as a restriction element for CD8 T cells [5], [6], [7], [8], [9]. However, the mechanisms underlying the development of CD8-mediated immunity remain uncertain. The induction of CMI mediated by CD8^+^ cells requires the presentation of antigens by specialized cells of the immune system named dendritic cells (DC) [15]. Studies using the *S.* Typhimurium mouse model have showed that DC can either directly (upon uptake and processing of *Salmonella*) or indirectly (by bystander mechanisms, including cross-presentation) present *Salmonella* antigens [16]. Cross-presentation denotes the ability of certain antigen-presenting cells, including DC, to acquire proteins from other tissue cells through endocytic mechanisms, and direct them into their own MHC I pathway to be subsequently presented to naïve T-cells [17], [18], [19], [20]. These events will result in proliferation and differentiation of naïve T-cells into memory cells, a process that is accompanied by changes in the expression of surface molecules [21].

Infection of susceptible mice with *S.* Typhimurium is considered a model for the pathogenesis of human typhoid fever [22]. However since *S.* Typhi infection is restricted to humans [22], it is not clear whether the conditions for DC maturation and/or patterns of antigen presentation induced in response to *S.* Typhimurium and *S.* Typhi infection are identical [22], [23]. In fact, previous studies have shown that antigen presenting cells from different animal species might present different sets of peptides [8]. To investigate how *S.* Typhi regulates the development of CD8^+^ CMI in humans, we examined the effects of *S.* Typhi on the maturation of DC and on their ability to prime naive CD8^+^ T cell responses. We report here that DC, through suicide cross-presentation, uptake apoptotic *S.* Typhi-infected human cells and release IFN-γ and IL-12p70, leading to the subsequent presentation of bacterial antigens and triggering the induction of mostly CD3^+^CD8^+^CD45RA^−^CD62L^−^ memory T cells. This study is, to our knowledge, the first demonstration of the effects of *S.* Typhi on human DC maturation and on their ability to prime CD8^+^ cells and highlights its significance in eliciting adaptive immunity to *S.* Typhi.

## Results

### *S.* Typhi drives DC maturation

**Figure 1:**
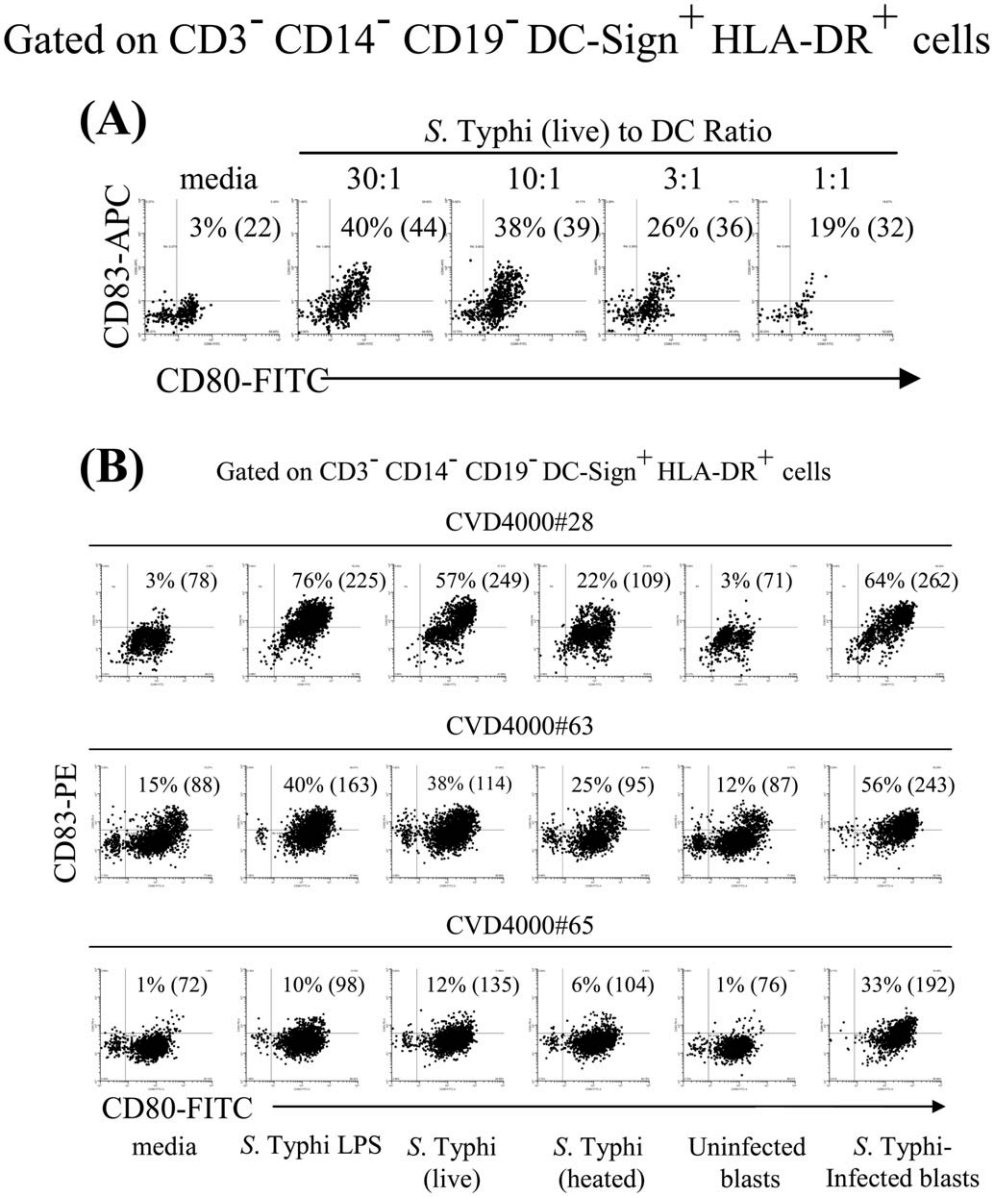
Modulation of cell surface expression of CD80 and CD83 molecules during *S.* Typhi-induced DC maturation. Immature DC were cultured for 30 h in the absence (media) or in the presence of LPS (1 µg/ml), with live or heat-killed *S.* Typhi at different MOI or uninfected or *S.* Typhi-infected blasts at a DC∶blast ratio of 4∶1. Cells were stained with mAbs to CD3, CD14, CD19, DC-Sign, HLA-DR, CD80 and CD83 or isotype-matched control Abs and analyzed by flow cytometry. Histogram shows the levels of CD80 and CD83 expression on CD3^−^ CD14^−^CD19^−^ DC-Sign^+^ HLA-DR^+^ gated cells. Numbers correspond to the % of CD80^+^ CD83^+^ positive cells in the indicated quadrant in each histogram followed by mean fluorescence intensity (MFI) of all CD80 positive cells (in parenthesis). Panel “A” shows the results of one volunteer, CVD5000#12U. Panel “B” shows the results of three volunteers, CVD4000# 28, 63 and 65.

To efficiently present antigens to naïve T cells, immature DC must be activated to mature, a process accompanied by up-regulation of MHC and costimulatory molecules [24]. To investigate whether *S.* Typhi drives DC maturation, we evaluated the DC surface expression of CD80 and CD83 costimulatory molecules after different stimulatory conditions. Unstimulated DC (media) were used as negative controls. We observed that DC pulsed with live *S.* Typhi increased their surface expression of CD80 and CD83 in a dose-dependent manner (Fig. 1A) with levels comparable to those induced following exposure to LPS, a potent DC maturation factor [25] (Fig. 1B). Increases, albeit at lower levels, were also apparent when DC were pulsed with heat-inactivated *S.* Typhi demonstrating that neither bacterial infection nor viability was required for the observed effects. These results are in agreement with others using *S.* Typhimurium in a mouse model [26]. However, the exposure of DC to *S.* Typhi-infected blasts induced comparable or higher levels of CD80 and CD83 expression on DC than those induced by exposure of DC to live *S.* Typhi or LPS (Fig. 1B). Of interest, the expression level of CD80 and CD83 molecules on DC pulsed with not-infected blasts were similar to those observed in DC cultured in media alone (Fig. 1B). In conclusion, the appearance of DC exhibiting higher levels of CD83 and CD80 molecules after incubation with *S.* Typhi antigens demonstrated that *S.* Typhi drives DC maturation in humans.

### *S.* Typhi stimulation augments the ability of DC to generate pro-inflammatory cytokines *in vitro*

**Figure 2:**
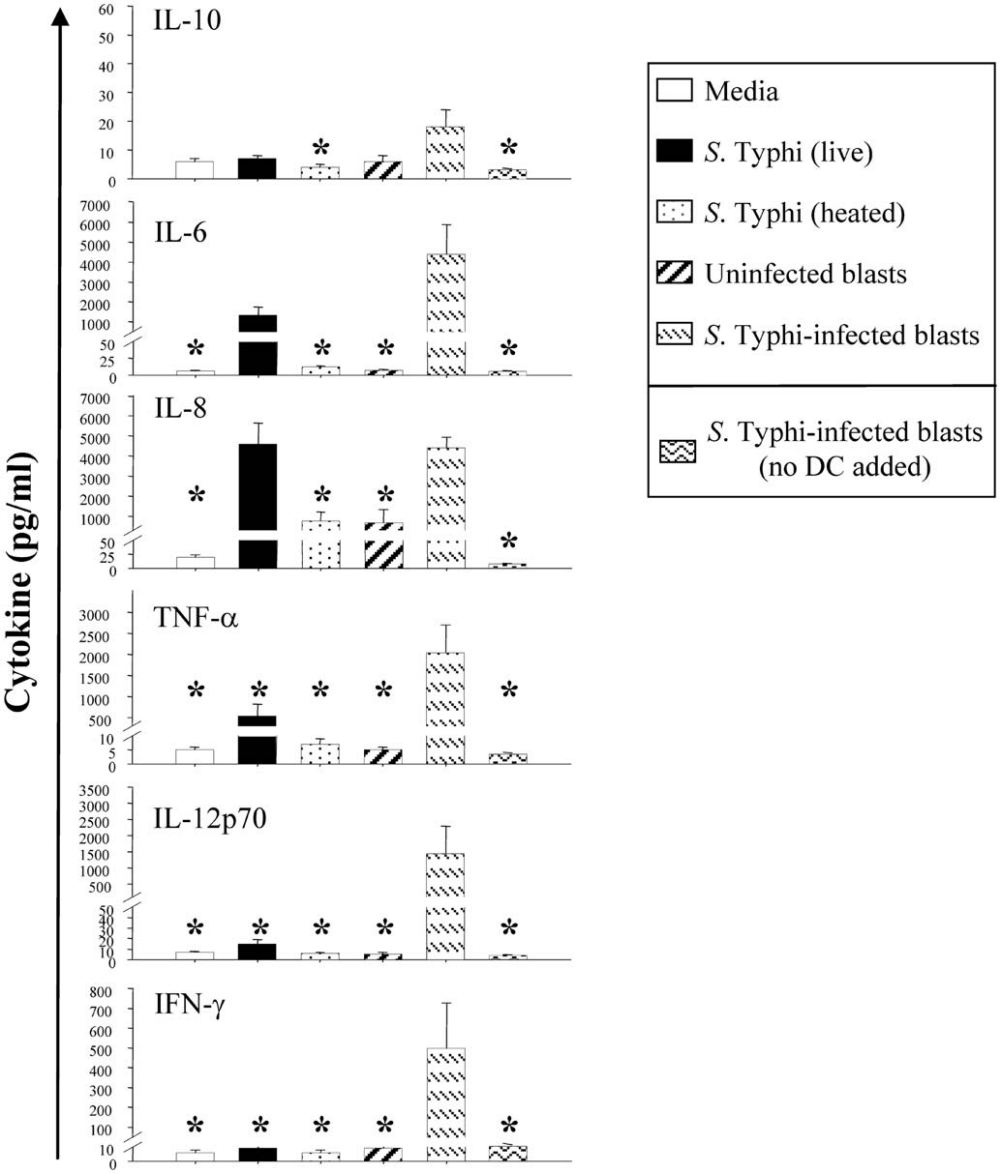
*S.* Typhi-infected blasts are the most effective stimulus in inducing DC to secrete pro-inflammatory cytokines *in vitro*. Immature DC were cultured in the absence (media) or in presence of live or heat-killed *S.* Typhi at a MOI of 10∶1, uninfected or *S.* Typhi-infected autologous blasts at DC∶blast ratio of 4∶1. *S.* Typhi-infected blasts only (without adding DC) were included as controls. After 48 h the supernants were harvested and cytokine production was measured using the flow cytometry-based BD cytometric bead array (CBA) assay. Bar graphs show mean + SE of 6 experiments using 5 different donors (*, p<0.05 compared with DC pulsed with *S.* Typhi-infected cells).

Another hallmark of DC maturation is the production of cytokines, such as IL-12 and IFN-γ, which are critical for the induction of CMI [27]. Because IL-12 and IFN-γ are essential for resistance to *Salmonella* infection in mice [28], [29], as they are likely to be in humans [5], [6], [9], [30], [31], it was of importance to evaluate the effect of *S.* Typhi on cytokine production by DC. To this end, DC were pulsed with heat-killed *S.* Typhi or wild-type *S.* Typhi at a 10∶1 MOI, uninfected or *S.* Typhi-infected blasts at a 1∶4 blast to DC ratio during 48 hours. DC pulsed with heat-killed or live *S.* Typhi produced high levels of pro-inflammatory cytokines such as IL-6, IL-8 and TNF-α but only low levels or no IL-12 p70 (Fig. 2). This low or lack of IL-12 secretion does not seems to be the result of IL-10 production by these cells. DC pulsed with heat-killed or live *S.* Typhi elicited levels of IL-10 only slightly different than those detected in culture supernatants of DC incubated in medium alone (Fig. 2).

Although it has been shown that DC can produce concomitantly IFN-γ and IL-12 [32], in our culture conditions, i.e., when DC were pulsed either with heat-killed *S.* Typhi or with live *S.* Typhi, IFN-γ was below the level of sensitivity of the CBA assay (Fig. 2). Surprisingly, a cytokine pattern for efficient T cell priming (i.e., high levels of IL-12 p70 and IFN-γ) was only observed after co-culture of DC with *S.* Typhi-infected blasts (Fig. 2). A low ratio of S. Typhi-infected cells per DC (1∶4) was sufficient to concurrently stimulate high levels of IL-12 p70 and IFN-γ. This phenomenon was found to be dose-dependent (data not shown) and observed in all 5 subjects studied. Minimal or no cytokine production was detected in control cultures with *S.* Typhi-infected blasts alone, i.e., in the absence of DC (Fig. 2). These results suggested that IL-12 p70 and IFN-γ production is a function of uninfected DC being stimulated by *S.* Typhi antigens from infected blasts. Moreover, this is the first demonstration of IL-12 p70 production by DC upon stimulation with *Salmonella* antigens.

### *S.* Typhi induces apoptosis in human blasts and DC

**Figure 3:**
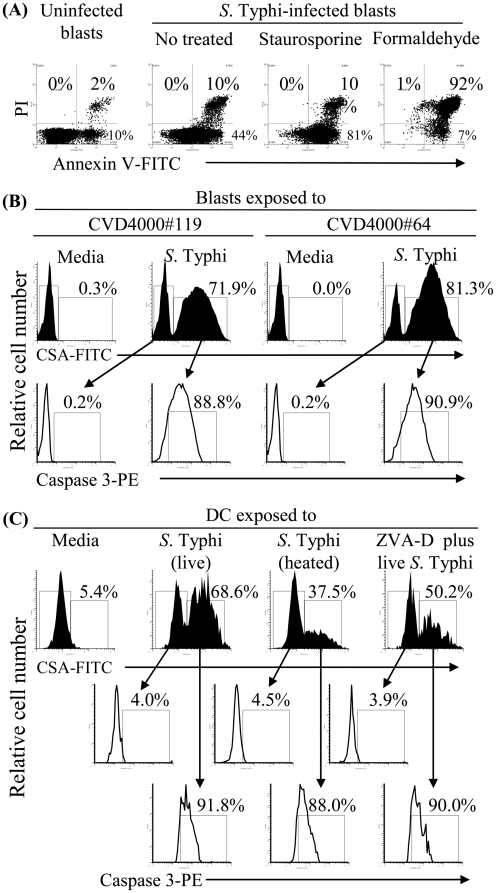
*S.* Typhi induces apoptosis in human cells. Blasts or DC were infected with *S.* Typhi at a MOI of 10∶1 and analyzed for apoptosis 3 hours after infection. DC were also exposed to heated-killed *S.* Typhi or treated with ZVA-D. Uninfected cells (media) were used as negative controls. Cells treated with staurosporine (8 µg/ml) or 1% formaldehyde were used as positive controls for apoptosis and necrosis respectively. Cell apoptosis was monitored by Annexin V and PI fluorescent dyes and by staining cells for cleaved caspase-3. (A) Blasts from volunteer A00302-5008 were stained with FITC-annexin V and PI or isotype-matched control antibodies and analyzed by flow cytometry. (B) Blasts from volunteers CVD4000#119 and CVD4000#64 and (C) DC from volunteer CVD4000#64 were surface stained for caspase-3 and *Salmonella* common structural antigens (CSA). Numbers correspond to the percentage of positive cells in the indicated quadrants or regions in each histogram. Arrows in panels B and C depict the expression of caspase 3 on cells expressing, or not, CSA.

Based on the above observations, we next investigated the role of apoptosis in the generation of antigens that could then be acquired by uninfected DC. It is known that bacteria-induced apoptotic cells can serve as a reservoir of antigens for cross-presentation by DC [16]. Previous studies have shown that some *Salmonella* serovars, including *S.* Typhimurium and *S.* Typhi, can induce death in infected macrophages by apoptotic mechanisms [33], [34]. Here, cell apoptosis was monitored by Annexin V and PI fluorescent dyes and by staining cells for cleaved caspase-3. Blasts were infected with *S.* Typhi at MOI of 10∶1 and analyzed for apoptosis after 3 hours of incubation. Uninfected blasts were used as negative controls. Cells treated with staurosporine or formaldehyde were used as positive controls for apoptosis and necrosis, respectively. Cells were stained with Annexin V and PI dyes or anti-caspase-3 antibody as well as isotype-matched control mAbs and analyzed by flow cytometry. We observed that *S.* Typhi-infected blasts undergo increased apoptosis, as detected by annexin V binding in the absence of PI staining, as compared to uninfected blasts (media) (Fig. 3A). Caspase-3-positive cells were also detected in *S.* Typhi-infected blasts (Fig. 3B). Both methods of detection identified similar percentages of apoptotic cells in *S.* Typhi-infected blasts. Similarly, we observed that DC pulsed with live *S.* Typhi increased apoptosis, as detected by caspase-3 staining (Fig. 3C). Increases, albeit at lower levels, were also apparent when DC were pulsed with heat-inactivated *S.* Typhi (Fig. 3C). Apoptosis was inhibited ∼30% when the incubation was carried out in the presence of ZVA-D, a powerful, irreversible and cell permeable inhibitor for caspases (Fig. 3C). Together these results further support the possibility that *Salmonella*-induced apoptosis is responsible for loading uninfected DC with *S.* Typhi antigen.

### DC efficiently uptake *S.* Typhi-antigens triggering a suicide mechanism

**Figure 4:**
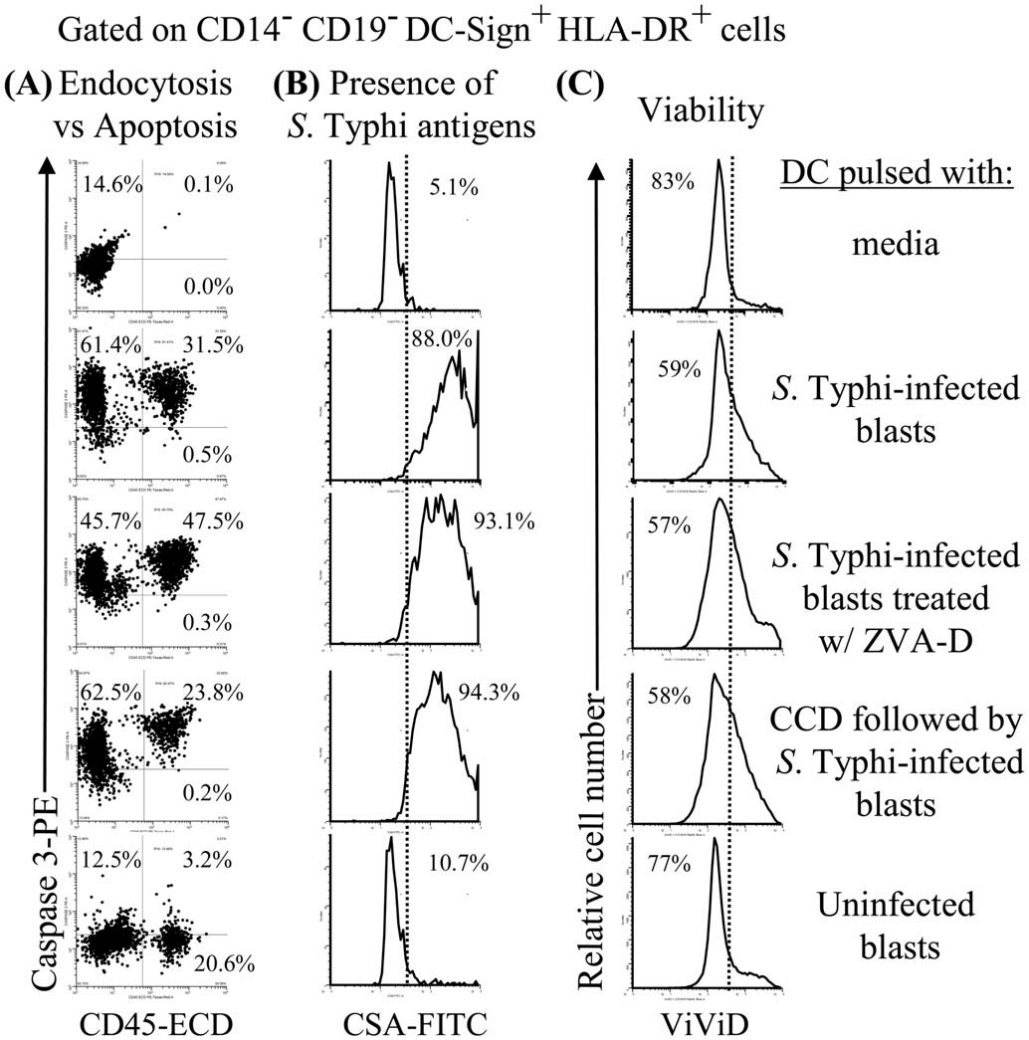
DC efficiently uptake *S.* Typhi-infected blasts. DC from volunteer CVD4000#64 treated or not with ZVA-D or CCD agents were incubated with uninfected or *S.* Typhi-infected CD45-labeled blasts at a DC∶blast ratio of 1∶5 at 37°C. After 2 hours of incubation, DC were stained with ViViD, followed by surface staining with mAbs to HLA-DR, DC-Sign, *Salmonella* common structural antigens (CSA) and caspase-3 and analysed by flow cytometry. DC were gated based on their scatter characteristics. Single DC were selected by gating on forward scatter height vs. forward scatter area and then on HLA-DR and DC-Sign. (A) endocytosis and apoptosis on DC was analyzed by studying expression of CD45 and caspase-3, respectively. (B) The presence of *S.* Typhi antigens on caspase-3+ cells were analyzed by determining their expression of CSA. (C) Viability of DC under different culture conditions was evaluated by ViViD as a dead cell exclusion marker. Dotted lines represent the cut-offs between positive and negative cells. Numbers correspond to the percentage of positive cells in the indicated quadrants or regions in each histogram. These results are representative of 1 of 3 volunteers with similar results. Additional data is provided in Fig. 5.

**Figure 5:**
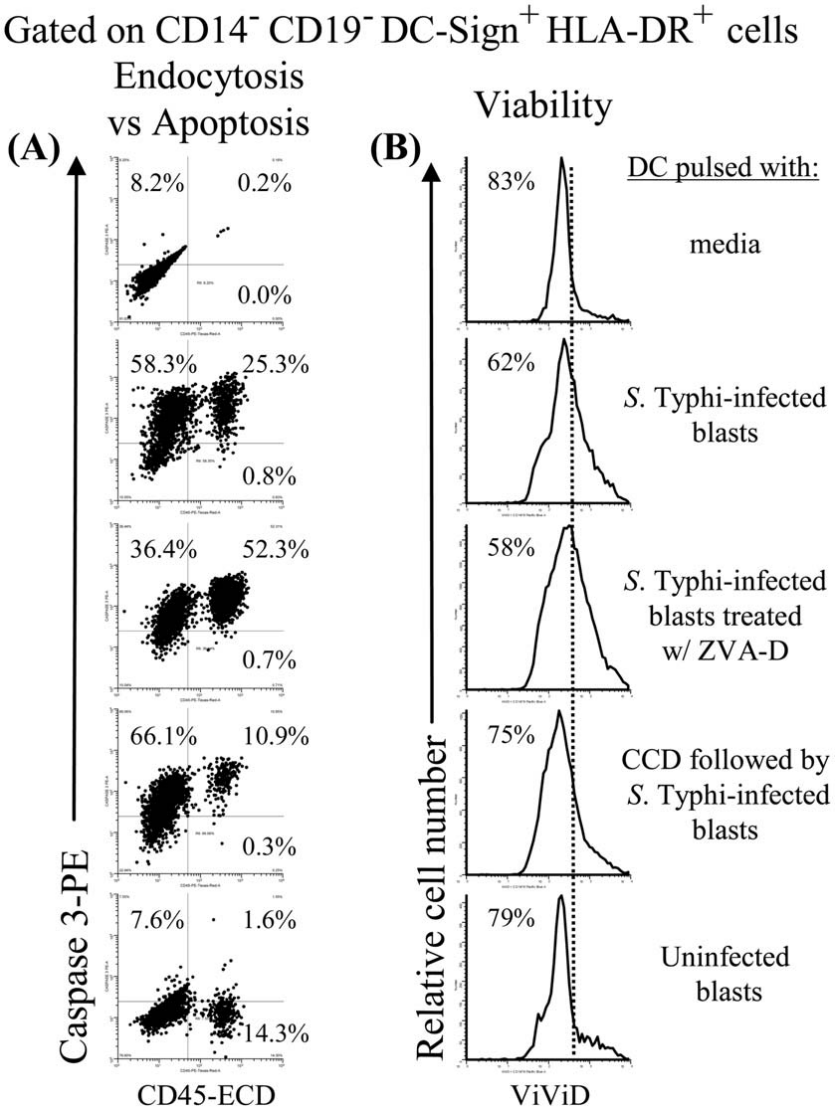
DC efficiently uptake *S.* Typhi-infected blasts. DC from volunteer CVD4000#119 treated or not with ZVA-D or CCD agents were incubated with uninfected or *S.* Typhi-infected CD45-labeled blasts at a DC∶blast ratio of 1∶5 at 37°C. After 2 hours of incubation, DC were stained with ViViD, followed by surface staining with mAbs to HLA-DR, DC-Sign, *Salmonella* common structural antigens (CSA) and caspase-3 and analysed by flow cytometry. DC were gated based on their scatter characteristics. Single DC were selected by gating on forward scatter height vs. forward scatter area and then on HLA-DR and DC-Sign. (A) endocytosis and apoptosis on DC was analyzed by studying expression of CD45 and caspase-3, respectively. (B) Viability of DC under different culture conditions was evaluated by ViViD as a dead cell exclusion marker. Dotted lines represent the cut-offs between positive and negative cells. Numbers correspond to the percentage of positive cells in the indicated quadrants or regions in each histogram. These results are representative of 1 of 3 volunteers with similar results.

To directly demonstrate the ability of DC to uptake *S.* Typhi-infected blasts, we incubated them with infected blasts labeled with a mAb to CD45, a surface marker present in all human leukocytes. After a 2 hour incubation, an average of 20% of the DC that were incubated with *S.* Typhi-infected blasts were positive for CD45, indicating capture of blast-derived material (Figs. 4A and 5A represent 2 out of 3 experiments using 3 different subjects). Capture was inhibited by 25–57% in the various repeat experiments when the incubation was carried out in the presence of CCD, an inhibitor of phagocytosis (Figs. 4A and 5A). To exclude the possibility that the decrease in the capture of *S.* Typhi-infected blasts was due to the direct toxic effect of CCD on DC, we measured the viability of the DC in presence and absence of CCD. To this end, we stained DC with ViViD, an amine-reactive fluorescent dye used to evaluate mammalian cell viability by flow cytometry. As shown in Figures 4C and 5B, similar DC viabilities were observed when DC were cultured with *S.* Typhi-infected blasts in the presence or absence of CCD.

Despite the fact that at the beginning of the cultures the numbers of the DC loaded with either *S.* Typhi or infected-blasts were the same, we can not rule-out the possibility that as the cultures progress, there is an increase in DC death after *S.* Typhi infection when compared to those cultured with infected-blasts. In this case, having fewer DC might adversely impact the cytokines production by *S.* Typhi-infected DC. To address this issue, we measured the apoptosis and viability of DC cultured with either *S.* Typhi or *S.* Typhi-infected blasts. After 2 hours of incubation, we compared the percentage of cells stained with anti-caspase-3 and ViViD by flow cytometry. As demonstrated by caspase-3 staining, DC cells exposed to *S.* Typhi had equal or less apoptosis than DC exposed to *S.* Typhi-infected blasts (Fig. 3C and Figs. 4A and 5A). Of note, the presence of *S.* Typhi antigens was detected on virtually all caspase-3+ cells by *Salmonella* common structural antigens (CSA) staining (Fig. 4B). DC cells exposed to *S.* Typhi (data not shown) had equal or higher viability than DC exposed to *S.* Typhi-infected blasts (Figs. 4C and 5B). Interestingly, not all caspase-3^+^ DC were double positive for CD45, showing that the caspase-3 mediated apoptosis was not due to preferential uptake of infected-blast cells. This might occur as the result of a bystander effect in which DC undergoing apoptosis following phagocytosis of *S.* Typhi-infected blasts might signal some of the neighboring DC to rapidly engulf them leading these DC to also undergo apoptosis. This would offer a mechanism for efficiently making additional *Salmonella* antigens available to new DC. This hypothesis is consistent with previous work showing that in mice *Salmonella* can be cytotoxic for liver phagocytes, whether these phagocytes harbored intracellular bacteria or not [35], [36]. Moreover, while the addition of CCD decreased the amount of DC loaded with CD45-infected blasts, no changes were observed in the caspase-3^+^ levels of the CD45-negative DC population. Interestingly, when DC were cultured with *S.* Typhi-infected blasts pre-treated with the apoptosis inhibitor ZVA-D, the percentage of caspase-3^+^ DC double positive for CD45 increased from 34–52% while the general apoptosis level, as well as their viability remained unchanged (Figs. 4 and 5). These results suggest: (a) an irreversible induction of apoptosis after ingesting *S.* Typhi–infected cells and (b) that the cross-presentation by DC might be related to *S.* Typhi-antigen itself rather than by the apoptotic nature of the *S.* Typhi-infected blasts. Thus, the apoptosis elicited by *S.* Typhi antigens can be the result of at least two pathways leading to cross-presentation: (a) through a bystander effect on neighboring DC and (b) through phagocytosis of *S.* Typhi–infected blasts which leads to the dead of these DC via irreversible apoptosis (i.e., a “suicide mechanism”). In this way, having more *S.* Typhi-infected blasts engulfed by DC might result in more *Salmonella* antigens available for cross-presentation resulting from more DC undergoing apoptosis.

### Generation of cytokine-producing effector cells

**Figure 6:**
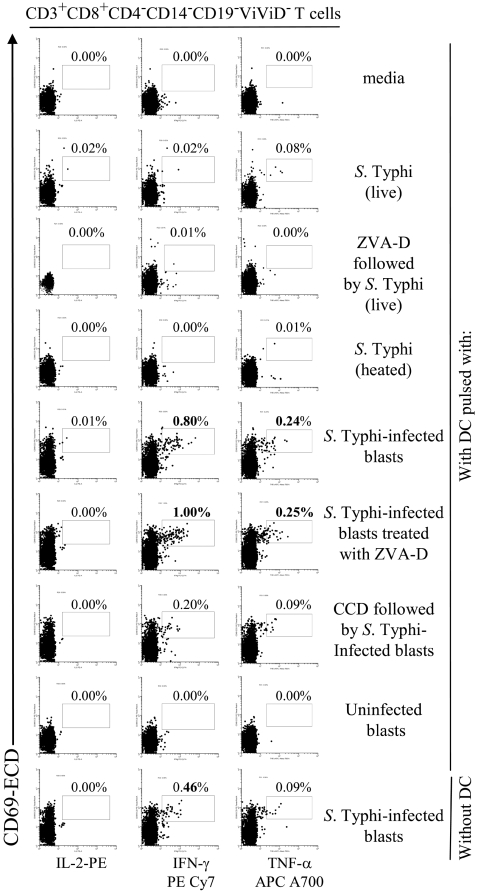
DC priming of *S.* Typhi-specific T cell responses. PBMC from volunteer CVD4000#63 were co-cultured with DC alone (media), or pre-mixed with live or heat-killed *S.* Typhi at a MOI of 10∶1, or uninfected or *S.* Typhi-infected blasts at a 1∶1 blast∶DC ratio. In some cases, DC were pre-treated with ZVA-D or CCD before exposure to *S.* Typhi or *S.* Typhi-infected blasts respectively. In other cases, blasts were treated with ZVA-D before co-culture with DC. After 20 hours of incubation, cells were surface stained with a combination of mAb to CD3, CD4, CD8, CD14 and CD19 as well as ViViD. After fixation and permeabilization, cells were intracellularly stained for IL-2, IFN-γ, TNF-α and CD69 and analyzed by multichromatic flow cytometry. Lymphocytes were gated based on their light scatter characteristics. Single lymphocytes were gated based on forward scatter height vs. forward scatter area. A “dump” channel was used to eliminate dead cells (ViViD^+^) as well as CD14^+^ and CD19^+^ cells from analysis. This was followed by additional gating on CD3, CD4 and CD8, to identify cytokine-producing CD8^+^ T cells. Each cytokine was gated individually. Numbers correspond to the percentage of positive cells in the indicated regions in each histogram. These results are representative of 1 of 4 volunteers with similar results. Additional data is provided in Fig. 7.

**Figure 7:**
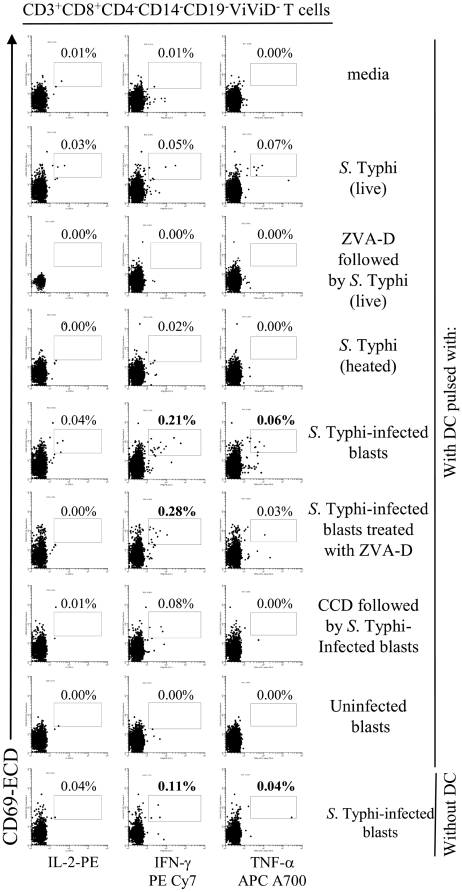
DC priming of *S.* Typhi-specific T cell responses. PBMC from volunteer CVD4000#65 were co-cultured with DC alone (media), or pre-mixed with live or heat-killed *S.* Typhi at a MOI of 10∶1, or uninfected or *S.* Typhi-infected blasts at a 1∶1 blast∶DC ratio. In some cases, DC were pre-treated with ZVA-D or CCD before exposure to *S.* Typhi or *S.* Typhi-infected blasts respectively. In other cases, blasts were treated with ZVA-D before co-culture with DC. After 20 hours of incubation, cells were surface stained with a combination of mAb to CD3, CD4, CD8, CD14 and CD19 as well as ViViD. After fixation and permeabilization, cells were intracellularly stained for IL-2, IFN-γ and TNF-α and analyzed by multichromatic flow cytometry. Lymphocytes were gated based on their scatter characteristics. Single lymphocytes were gated based on forward scatter height vs. forward scatter area. A “dump” channel was used to eliminate dead cells (ViViD^+^) as well as CD14^+^ and CD19^+^ cells from analysis. This was followed by additional gating on CD3, CD4 and CD8, to identify cytokine-producing CD8^+^ T cells. Each cytokine was gated individually. During sample acquisition, routinely 300,000-500,000 events were collected in the forward and side scatter (FS/SS) lymphocyte gate. This large number of gated lymphocyte events was necessary to ensure that a sufficient number of positive cells for a defined subset would be collected for each tube analyzed. Numbers correspond to the percentage of positive cells in the indicated regions in each histogram. These results are representative of 1 of 4 volunteers with similar results.

The effect of *Salmonella* on DC maturation and cytokine production described above, as well as their capacity to induce apoptosis in the infected cells, prompted us to investigate the ability of *Salmonella* antigen-loaded DC to prime naïve human T cells. Previous studies have shown that by exposing DC to antigen and then to naïve T cells *in vitro*, it is possible to mimic the process of antigen presentation *in vivo* [37], [38]. We used this approach to evaluate the ability of DC to either directly (e.g., priming upon uptake and processing of *Salmonella*) or indirectly (e.g., by cross-priming through a bystander mechanism) present *Salmonella* antigens to naïve T cells. This was studied by co-culturing *ex vivo* PBMC from individuals non-exposed to *S.* Typhi (naïve subjects) with DC that had been pulsed with live or heat-killed *S.* Typhi at 10∶1 MOI (priming), or pulsed with uninfected or *S.* Typhi-infected blasts at a 1∶1 blast to DC ratio (cross-priming). After 30 hours of incubation, the *S.* Typhi specificity of primed PBMC was evaluated by their ability to secrete IL-2, IFN-γ and TNF-α detected as measured by intracellular staining. To avoid infection of DC by live bacteria from dying infected-blasts, blasts were harvested, washed 3 times with RMPI containing gentamicin (100 µg/ml) and incubated for 1 hour at 37°C to kill and remove extracellular bacteria before being added to PBMC. Moreover, to assess the pre-existing background from cross-reactive effector T cells, a control consisting of PBMC co-cultured with the *S.* Typhi-infected blasts in the absence of DC was also included. Although PBMC in the presence of *S.* Typhi-infected blasts alone (in the absence of DC) produced moderate levels of cytokines, these levels were considerably lower than those observed in PBMC co-cultured with DC pre-mixed with *S.* Typhi-infected blasts (Figs. 6 and 7). Thus, these results clearly indicate that DC function as antigen-presenting cells. Four volunteers were studied to assess the relative importance of priming and cross-presentation in the early stages of *S.* Typhi infection. Results indicate different levels of cytokine production in the various volunteers. Whilst in 2 of 4 volunteers higher induction of cytokine production was observed following DC co-cultured with *S.* Typhi-infected blasts (Figs. 6 and 7), a third volunteer showed similar levels when PBMC were co-cultured DC exposed to live *S.* Typhi or with *S.* Typhi-infected blasts (data not shown), and in the 4^th^ volunteer the cytokine production was higher in cultures of PBMC with DC infected with live *S.* Typhi (data not shown). Therefore it is likely that both mechanisms, cross- and direct-priming by DC, might be involved in stimulating cytokine production from PBMC.

To confirm that DC are engulfing infected-blasts and subsequently processing them intracellularly, uninfected DC pre-treated with CCD and co-cultured with infected blasts were used as antigen-presenting cells in *ex vivo* PBMC cultures. As expected, CCD markedly inhibited the DC's ability to stimulate cytokine production by naïve PBMC in all 4 volunteers evaluated (Figs. 6 and 7).

To assess the role of apoptosis in antigen presentation we compared whether blocking apoptosis affected the ability of DC to stimulate T cells. To this end, uninfected DC pre-treated or not with ZVA-D and co-cultured with live *S.* Typhi or infected blasts pre-exposed or not to ZVA-D were used as antigen-presenting cells in *ex vivo* PBMC from individuals non-exposed to *S.* Typhi (naïve subjects). A substantial decrease in cytokine production by PBMC was observed when DC were pre-treated with ZVA-D before exposure to live *S.* Typhi (4 of 4 volunteers) (Figs. 6 and 7, and data not shown). In contrast, when DC from the same volunteers were exposed to ZVA-D-pre-treated *S.* Typhi-infected blasts failed to affect cytokine production by PBMC (Figs. 6 and 7, and data not shown). While these observations support an important role for the induction of apoptosis in antigen presentation, they also suggest that apoptotic material from different origins might also contribute to this process to varying degrees. These results reinforce the idea that both mechanisms, cross- and direct-priming by DC are important in the development of *S.* Typhi-immunity.

### *S.* Typhi-specific memory CD8^+^ T cell subsets are expanded following priming by DC

**Figure 8:**
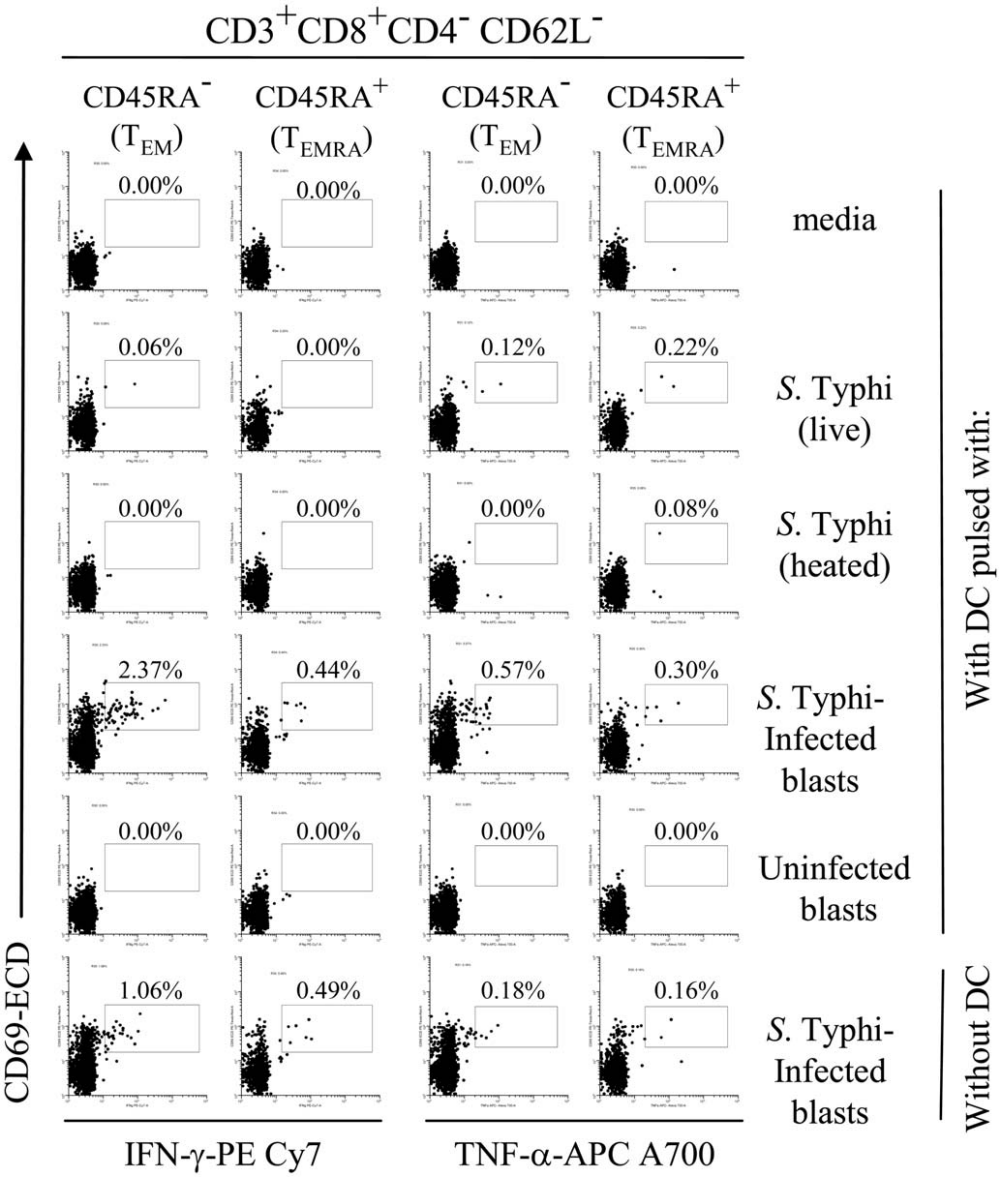
Induction of IFN-γ- and TNF-α-secreting CD8^+^ T cell subpopulations upon DC priming. PBMC from volunteer CVD4000#63 were co-cultured with DC alone (media), or pre-mixed with live or heat-killed *S.* Typhi at a MOI of 10∶1, or uninfected or *S.* Typhi-infected cells at a 1∶4 blast∶DC ratio. After 20 hours of incubation, cells were surface stained with a combination of mAb to CD3, CD4, CD8, CD14, CD19, CD45RA, and CD62L as well as ViViD. After fixation and permeabilization, cells were intracellularly stained for IFN-γ and TNF-α and analyzed by multichromatic flow cytometry. Lymphocytes gated as described in Figure 5 were followed by additional gating on CD3, CD4 and CD8, as well as CDR45RA versus CD62L to analyze T_EM_ and T_EMRA_ cell subsets. Numbers correspond to the percentage of positive cells in the indicated regions in each histogram. These results are representative of 1 of 4 volunteers with similar results. Additional data is provided in Fig. 9.

**Figure 9:**
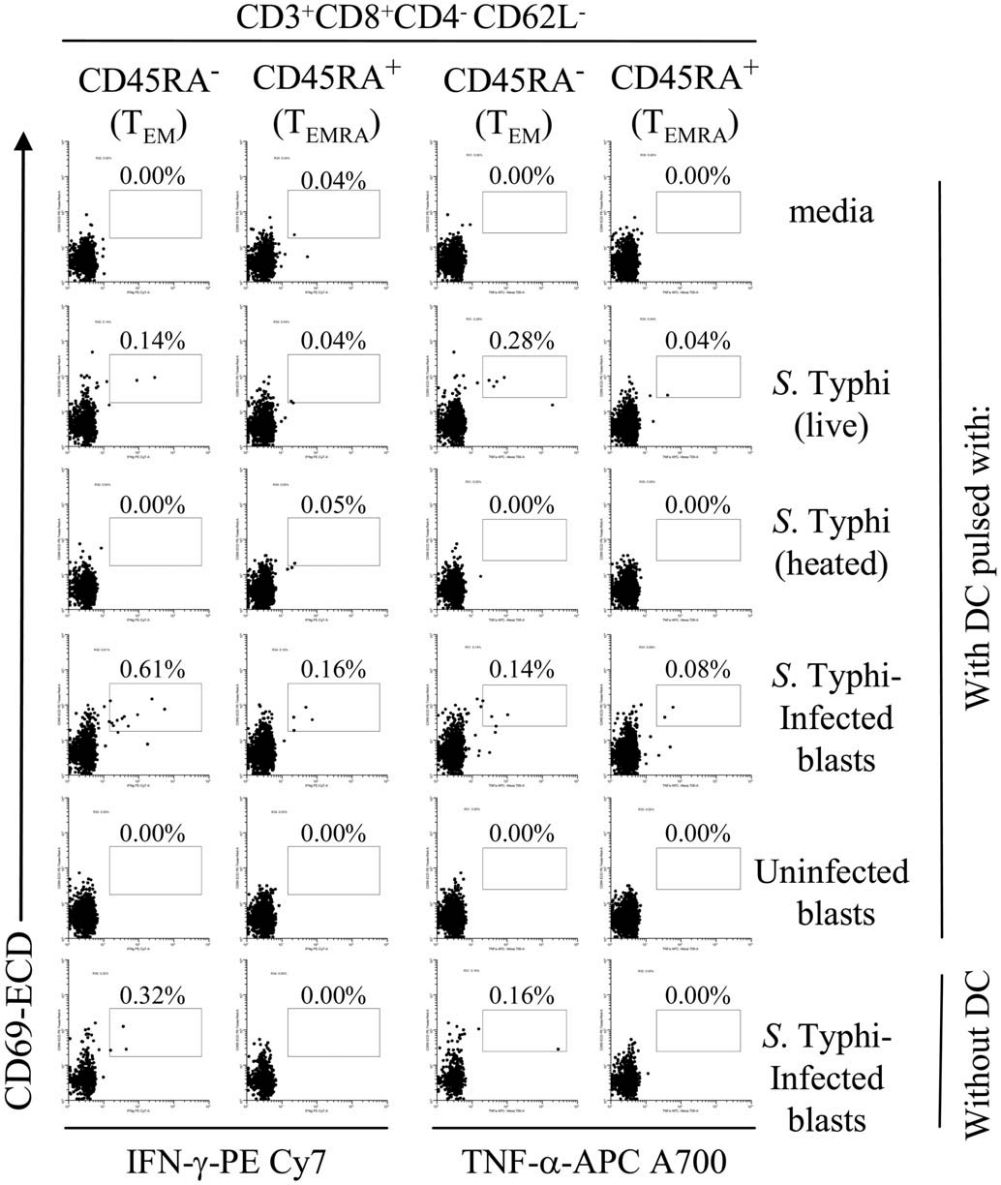
Induction of IFN-γ- and TNF-α-secreting CD8^+^ T cell subpopulations upon DC priming. PBMC from volunteer CVD4000#65 were co-cultured with DC alone (media), or pre-mixed with live or heat-killed *S.* Typhi at a MOI of 10∶1, or uninfected or *S.* Typhi-infected cells at a 1∶4 blast∶DC ratio. After 20 hours of incubation, cells were surface stained with a combination of mAb to CD3, CD4, CD8, CD14, CD19, CD45RA, and CD62L as well as ViViD. After fixation and permeabilization, cells were intracellularly stained for IFN-γ and TNF-α and analyzed by multichromatic flow cytometry. Lymphocytes gated as described in Figure 5 were followed by additional gating on CD3, CD4 and CD8, as well as CDR45RA versus CD62L to analyze T_EM_ and T_EMRA_ cell subsets. Numbers correspond to the percentage of positive cells in the indicated regions in each histogram. These results are representative of 1 of 4 volunteers with similar results.

We next investigated which CD8 cell populations within PBMC preferentially produced IFN-γ and TNF-α cytokines under the different stimulatory conditions described above. We evaluated the induction of cytokine production in the various memory CD8^+^ T cell subsets, defined as follows: central memory T cells (T_CM_, CD45RA^-^CD62L^+^), effector memory T cells (T_EM_, CD45RA^−^CD62L^−^) and naïve T cells (T_n_, CD45RA^+^CD62L^+^) [14]. We observed that the majority of CD8^+^ T cells elicited were composed of classical T_EM_ (Figs. 8 and 9). Minor increases were also observed in T_EMRA_. No significant increases were observed in T_CM_ and T naïve subsets (data not shown).

**Figure 10:**
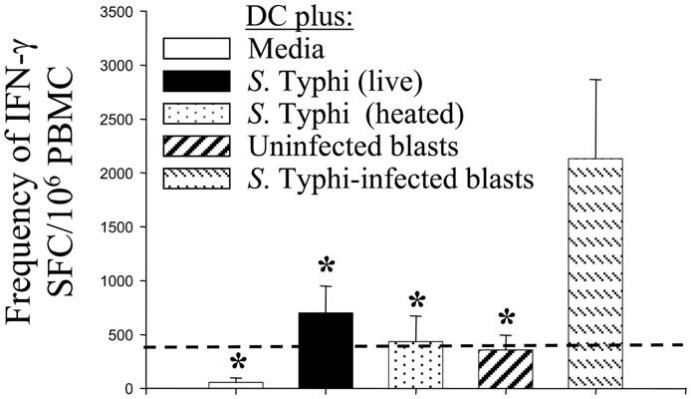
Cross-priming DC are the most effective stimulus in promoting the expansion of *S.* Typhi-specific T cells. Net frequencies of IFN-γ-spot forming cells (SFC) were assessed by an IFN-γ ELISPOT assay using *in vitro* expanded PBMC as effectors and autologous blasts infected with live *S.* Typhi as stimulators. PBMC were co-cultured with their own DC alone or DC that had been pulsed with live or heat-killed *S.* Typhi, or uninfected or *S.* Typhi-infected autologous blasts at a 1∶4 blast∶DC ratio. Net frequencies of IFN-γ SFC were calculated as described in Materials and Methods. The dashed line represents the cut-off for positive-ELISPOT assays determined as described in Materials and Methods. Bar graphs show means+SE of 3 experiments using 4 different donors (*, p<0.05 compared with DC pulsed with *S.* Typhi-infected cells).

We next examined the ability of primed T cells to expand. Eight to twelve days after co-culture of PBMC with DC under the various culture conditions discussed above, the frequency of *S.* Typhi-specificity of PBMC was evaluated by their capacity to secrete IFN-γ by ELISPOT. Significant increases in net frequencies of IFN-γ-SFC were observed between PBMC previously stimulated with DC pulsed with *S.* Typhi-infected cells and all other groups (Fig. 10). Although frequencies above the threshold of positive IFN-γ-SFC responses were observed in PBMC following stimulation with DC pulsed with live *S.* Typhi, no statistically significant differences were observed among these frequencies and the frequencies observed in cultures with heated *S.* Typhi, uninfected blasts and media controls (Fig. 10). Taken together, these observations suggest that DC cross-priming is very effective in promoting the expansion of *S.* Typhi-specific T cells.

## Discussion

Here we provide the first direct demonstration that DC, through suicide cross-presentation, uptake *S.* Typhi-infected human cells and release IFN-γ and IL-12p70, leading to the subsequent presentation of bacterial antigens and triggering the induction of mostly CD3^+^CD8^+^CD45RA^−^CD62L^−^ memory T cells.

We observed that upon infection with live *S.* Typhi, human DC produced high levels of pro-inflammatory cytokines IL-6, IL-8 and TNF-α, but low levels of IL-12 p70 and IFN-γ. In contrast, DC co-cultured with *S.* Typhi-infected cells produced high levels of IL-12 p70, IFN-γ and TNF-α. These interesting and novel findings suggest that these cytokines play a critical role in controlling cross-priming in *S.* Typhi infection in humans. Several lines of evidence support the contention that high IFN-γ and IL-12p70 production is a consequence of cross-presentation of *S.* Typhi antigens by DC rather than being exclusively associated with bacteria infection. For example, in previous studies, infection of DC with different *Salmonella* strains resulted in low levels of IL12p40 secretion, while the IL-12p70, biologically active form of IL-12 [39], was undetectable or only detected at levels slightly above those detected in culture supernatants of DC incubated in medium [16], [26], [39]. Additionally, challenge of DC with increasing amounts of apoptotic cells expressing ovoalbumin, a non bacterial antigen, resulted in substantial secretion of IL-1β, IL10 and TNF-α but not IL-12 p70 or IFN-γ [40]. It is unclear why DC co-cultured with *S.* Typhi-infected cells, but not directly infected with *S.* Typhi, produced high levels of IL-12 p70, IFN-γ and TNF-α. We speculate that IL-12 p70 production is induced by “cell-to-cell contact” model. This hypothesis is supported by recent findings showing that IL-12 p70 production by DC is induced by a concomitant contact of TLR and CD40 on the DC with LPS and CD40L on the T cells, respectively [41]. In our model, DC might interact with LPS from *S.* Typhi and CD40L, both of which are present in the membranes of *S.* Typhi-infected blasts. Further studies will be required to dissect the precise mechanisms underlying the effects of IFN-γ and IL-12p70 in cross-presentation.

Here, we also demonstrate that human DC directly (upon *Salmonella infection*) or by active phagocytosis of *S.* Typhi–infected cells (a suicide pathway) and/or bystander effect in neighboring DC are very efficient in priming T cells. These results resemble previous studies using the *S.* Typhimurium mouse model that demonstrated that DC can either directly (upon uptake and processing of *Salmonella*) or indirectly (by bystander mechanisms) present *Salmonella* antigens [16], [42]. Interestingly, we also found that the suicide pathway might be related to *S.* Typhi-antigen cross-presentation by DC rather than by the apoptotic nature of *S.* Typhi-infected blasts. This is consistent with a previous model showing that necrotic cells or even living cells were also cross-presented by human DC to CD8^+^ T cells [43]. Moreover, as shown in other systems, the capacity to act as bystander antigen presented cells appears to be a unique feature of DC, since bystander macrophages ingest Salmonella-induced apoptotic cells but are unable to present peptides from Salmonella antigens for T cell recognition [32]. We share the view of other investigators that both mechanisms are important and under specific circumstances one will predominate over the other [19]. In this regard, it will be important to study the role of the level and nature (e.g., dead or live cells, cell fragments) of antigen or environmental factors (e.g., induction of heat-shock proteins by stress prior to cell death) in favoring a particular mechanism [43]. For example, it has been shown that the level of antigen expressed by peripheral tissues must be relatively high to facilitate cross-presentation to naive CD8^+^ T cells. Below this level, peripheral antigens were unable to stimulate by cross-presentation and therefore ignored by naive CD8^+^ T cells [44].

We also demonstrate for the first time that DC pulsed with either live *S.* Typhi, or *S.* Typhi-infected blasts are effective in stimulating cytokine production by CD8^+^ T cells, mostly classical T_EM_. This is in agreement with our previous work showing the predominance of T_EM_ subsets after immunization with CVD 909 typhoid vaccine [14]. It should be emphasized that although cells and phenotypes may be elicited and may even increase in number or functional potential with exposure to *S.* Typhi antigens, these increases by themselves do not prove that they play a role in protection. However, since *S.* Typhi is a human restricted pathogen and there are significant barriers in performing challenges with wild type *S.* Typhi in humans, the system used here constitutes a reasonable approach which allowed us to observe the generation and expansion of T_M_ cell subsets that might be directly relevant for resistance to *S.* Typhi infection in humans.

In sum, although other mechanisms might be involved in presentation of *S.* Typhi antigens, this study serves as the first demonstration of the effects of *S.* Typhi on human DC maturation and of their ability to cross-prime CD8^+^ cells and highlights its significance in eliciting adaptive immunity to *S.* Typhi.

## Materials and Methods

### Ethics Statement

The human experimentation guidelines of the US Department of Health and Human Services and those of the University of Maryland, Baltimore, were followed in the conduct of the present clinical research. All blood specimens were collected from volunteers that participated in University of Maryland Institutional Review Board approved protocol numbers HP-00040025 and HP-00040022 that authorized the collection of blood specimens for the studies included in this manuscript.

### Volunteers

Thirteen healthy adult volunteers, between 24–53 years old (5 female and 8 male), recruited from the Baltimore-Washington area and University of Maryland at Baltimore campus, participated in this study. The PBMC were isolated from the blood of volunteers by density gradient centrifugation and cryopreserved in liquid N_2_. Volunteers were without any antibiotic treatment and had normal blood counts at the times of blood collection. Before blood collections, volunteers were explained the purpose of this study and signed informed consents.

### Monoclonal antibodies for surface staining

Cells were surface stained with mAbs to CD1a, CD8, CD14, CD56, CD62L, TCRαβ, TCRγδ, HLA-DR and DC-Sign (BD Pharmingen, San Diego, CA, USA), CD4, CD27, CD45, CD45RA, CD80, CD83 (Beckman-Coulter, Miami, FL), CD3, CD4, CD8, CD19, CD45, CD45RA and CD62L (Molecular Probes, Eugene, OR).

### Generation and infection of human DC

DC were generated by standard procedures from peripheral blood monocytes. The cells were used before or after further treatment with (a) live or (b) 60°C heated-inactivated wild-type *S.* Typhi strain ISP1820 (obtained from Dr. J. Nataro, Center for Vaccine Development), at different multiplicity of infection (MOI), (c) uninfected or (d) *S.* Typhi-infected autologous blasts at different blast to DC ratios, or (e) LPS from *Salmonella* Typhi (1 µg/ml, Sigma) for 30–48 hours. DC cultured in media alone were used as negative control.

To infect DC, cells were incubated for 3 hours at 37°C in RPMI (without antibiotics) in the presence of *S.* Typhi at a MOI of 10∶1 [5], [6], [8], [9]. After incubation, DC were harvested, washed 3 times with RMPI containing gentamicin (100 µg/ml) and incubated for 1 hour at 37°C to kill and remove extracellular bacteria.

The phenotype of DC was examined by multichromatic flow cytometry using mAbs to CD14, CD3, CD14, CD19, CD80 and CD83 cell surface antigens. DC gated on CD3^−^CD14^−^CD19^−^ cells (“dump” channel) DC-Sign^+^ HLA-DR^+^ were analyzed for the expression of CD80 and CD83 maturation makers.

### Endocytosis assays

DC endocytosis was measured by uptake of CD45 ECD-labeled blasts by flow cytometry. Fluorescent CD45-labeled blasts were cocultured with approximately 10^5^ DC at 5∶1 blast to DC ratio. After 2 hours of incubation at 37°C, mixed cells were washed twice with cold PBS to stop endocytosis, and stained with an amine reactive viability dye (ViViD)(Molecular Probe) as a dead cell exclusion marker, followed by surface staining with mAbs to HLA-DR and DC-Sign. Cells were then washed and fixed in cold 1% formalin. The quantitative uptake of CD45-labeled blasts by DC was determined by flow cytometry. DC that either bound together or very close to blast cells without internalizing them were identified by doublet discrimination and excluded from analysis (i.e., only single DC identified by gating on forward scatter height vs. forward scatter area were analyzed). In some experiments, DC were treated with cytochalasin D (CCD) (10 µg/ml, Sigma) or Z-Val-Ala-Asp-FMK (ZVA-D) (100 µM, Sigma) for 1 hour before and/or during co-culture with blasts. All surface staining was carried out at 4°C.

### Preparation of *S.* Typhi-infected blast cells as stimulators

Autologous blasts were generated following standard procedures [5], [6], [8], [9]. Briefly, blasts were obtained by incubating PBMC with 1 µg/ml phytohemagglutinin (PHA-L) for 24 hours. PHA-activated PBMC were then washed three times with RPMI 1640, and cultured with 20 IU/ml human recombinant interleukin-2 (rhIL2) (Boehringer Gmbh, Mannheim, Germany) for 5 to 6 days. PHA-stimulated PBMC were then henceforth called “blasts”. Blasts were γ-irradiated (4,000 rads) before being used as stimulators for ELISPOT assays or as a source of infected cells for co-culture with DC. To confirm that targets were infected with *S.* Typhi, cells were stained with anti-*Salmonella* common structural antigens (CSA-1)-FITC (KPL, Gaithersburg, MD, USA) and analyzed by flow cytometry [5], [6], [8], [9].

### Quantification of Cytokines by Cytometric bead array (CBA)

Cytokine levels of IFN-γ, IL-1β, IL-2, IL-5, IL-6, IL-8, IL-10, IL-12p70 and TNF-α by DC was measured by flow cytometry-based BD Cytometric bead array (CBA). Briefly, DC were cultured in the absence (media) or presence of the various culture conditions discussed above for 48 hours. The supernatants were then harvested and kept at -70°C until assayed. CBA assays were carried-out following the manufacturer's instructions. The levels of sensitivity for the various cytokines ranged from 2.5–20 pg/ml.

### Apoptosis assays

Cell apoptosis was monitored by two assays: (A) by measuring membrane changes by staining cells with annexin-V and propidium iodide (PI) (APOAF Annexin V-FITC Apoptosis detection kit, Sigma) and (B) by measuring cytosol changes following staining with a rabbit polyclonal antibody to caspase-3 PE (BD-Pharmingen). Annexin V and PI staining were performed following the manufacturer's recommendations.

### Priming assays

Priming assays were performed as previously described [37], [38] with slight modifications. PBMC from healthy *S.* Typhi-unvaccinated adult volunteers were co-cultured with autologous DC at PBMC to DC ratios of 15–20∶1. The *S.* Typhi PBMC specificity or priming was evaluated by their ability to secrete cytokines *ex vivo* or *in vitro* detected by intracellular staining or ELISPOT, respectively. DC were stimulated as described above for 30-48 hours before exposure to the PBMC.

### Intracellular staining

After 1–2 hours of stimulation with *S.* Typhi antigens, brefeldin-A (BFA) (2 µg/ml, Sigma), a protein transport blocker, was added to the PBMC. After 16–18 hours, PBMC were surface and intracellularly for IFN-γ, TNF-α, IL-2 (B-D Pharmingen) and CD69 (Beckman-Coulter) to perform up to 13-color stainings. Cells were then resuspended in fixation buffer (1% formaldehyde) and analyzed as soon as possible by flow cytometry on an LSR-II instrument (BD Biosciences).

### IFN-γ ELISPOT assay

IFN-γ ELISPOT assays were performed as previously described [5], [6], [8], [9]. Net frequencies of spot forming cells (SFC) were calculated as previously described [5], [6], [8], [9]. The cut-off for *in vitro* expanded effector cells was established as the frequency of SFC/10^6^ PBMC in co-cultures of effectors with not-infected targets + 2 SE.

### Statistical analysis

All tests were performed using SigmaStat software (version 3.10, SSPS Science software products, Chicago, IL). Comparisons between groups were performed by the One-way ANOVA test. Specifically, in flow cytometric experiments, a response was considered specific if the differential in the number of positive events between experimental and negative control (media only) cultures was significantly increased by Chi-square tests. *P* values<0.05 were considered significant.
